# Polymer-Derived Biosilicate^®^-like Glass-Ceramics: Engineering of Formulations and Additive Manufacturing of Three-Dimensional Scaffolds

**DOI:** 10.3390/ma14185170

**Published:** 2021-09-09

**Authors:** Fulden Dogrul, Paulina Ożóg, Martin Michálek, Hamada Elsayed, Dušan Galusek, Liliana Liverani, Aldo R. Boccaccini, Enrico Bernardo

**Affiliations:** 1 FunGlass–Centre for Functional and Surface Functionalized Glass, Alexander Dubcek University of Trenčín, Študentská 2, 911 50 Trenčín, Slovakia; fulden.dogrul@tnuni.sk (F.D.); paulina.ozog@tnuni.sk (P.O.); martin.michalek@tnuni.sk (M.M.); dusan.galusek@tnuni.sk (D.G.); 2 Department of Industrial Engineering, Università degli Studi di Padova, Via Marzolo 9, 35131 Padova, Italy; hamada.elsayed@unipd.it; 3 Institute of Biomaterials, University of Erlangen-Nuremberg, Cauerstraße 6, 91058 Erlangen, Germany; liliana.liverani@fau.de (L.L.); aldo.boccaccini@fau.de (A.R.B.); 4 Ceramics Department, National Research Centre, El-Bohous Street, Cairo 12622, Egypt; 5 Joint Glass Centre of the IIC SAS, TnUAD and FChFT STU, Študentská 2, 911 50 Trenčín, Slovakia

**Keywords:** Biosilicate^®^ glass-ceramic, polymer-derived ceramics, SiOC, additive manufacturing, direct ink writing, digital light processing

## Abstract

Silicone resins, filled with phosphates and other oxide fillers, yield upon firing in air at 1100 °C, a product resembling Biosilicate^®^ glass-ceramics, one of the most promising systems for tissue engineering applications. The process requires no preliminary synthesis of parent glass, and the polymer route enables the application of direct ink writing (DIW) of silicone-based mixtures, for the manufacturing of reticulated scaffolds at room temperature. The thermal treatment is later applied for the conversion into ceramic scaffolds. The present paper further elucidates the flexibility of the approach. Changes in the reference silicone and firing atmosphere (from air to nitrogen) were studied to obtain functional composite biomaterials featuring a carbon phase embedded in a Biosilicate^®^-like matrix. The microstructure was further modified either through a controlled gas release at a low temperature, or by the revision of the adopted additive manufacturing technology (from DIW to digital light processing).

## 1. Introduction

Preceramic polymers [1], especially in the form of polysiloxanes, known also as ‘silicones’, have been recently recognized as raw materials for a new generation of bioceramics, such as Ca-, Ca-/Mg- and Ca-/Zn- silicates [2,3,4,5,6,7]. In particular, silicone resins, embedding micro- and nano-sized fillers, consisting of carbonates, hydroxides or simple oxides may yield the desired silicate with excellent purity, at a generally lower temperature (900–1100 °C), offering interesting shaping possibilities [8,9,10,11,12,13]. The use of silicones as a silica source (after firing) may be considered as safe, also considering the direct application of these polymers in composites for tissue engineering [9].

Along with a vast range of other polymer-derived ceramics, such bioceramics can be prepared by well-established polymer-shaping processes, including direct foaming [8,11] and modern additive manufacturing technologies, including direct ink writing and digital light processing [8,10,14], before thermal treatment. Some constituents may even operate at different stages of the overall process. Borate and phosphate fillers, if supplied in a hydrated form, act as foaming agents for silicone-based mixtures, due to the low temperature of dehydration (<350 °C) and the consequent water vapour release [8]. Upon firing, they form a liquid, which favours the ionic interdiffusion and catalyses the formation of silicate phases [7,8]. Upon cooling, the liquid phase forms glass surrounding the silicate crystals, so that the final products resemble glass-ceramics [8].

The ceramic transformation of silicones is sensitive to the firing atmosphere [7]. When treated in a non-oxidative atmosphere, silicones are not transformed into amorphous silica, but into a silicon oxycarbide ceramic (SiOC). SiOC is recognized as a nano-composite with turbostratic carbon nano-sheets embedded in silica-based glass, featuring some Si–C bonds in the siloxane network [15]. The reaction with suitable fillers may yield silicate/carbon composites. Fu et al. [16] and Zhu et al. [17], as an example, studied silicone/filler mixtures, which were used as pastes for direct ink writing of three-dimensional scaffolds, which transformed into composites featuring larnite (Ca_2_SiO_5_) or forsterite (Mg_2_SiO_5_), together with pyrolytic carbon. The carbon phase may enhance osteogenic differentiation [18] and, above all, cause a photo-thermal effect, by the absorption of infrared light. The heating of scaffolds can be an advantage utilized for cancer therapy or disinfection [19,20,21,22].

The fundamental objective of the present paper is to fully disclose the potential of direct ink writing of silicone-based inks in producing three-dimensional composite scaffolds with a well-defined composition, mimicking Biosilicate^®^ glass-ceramics. Such materials represent excellent alternatives to 45S5 Bioglass^®^ in bone tissue engineering applications [23,24,25]. More precisely, the investigation concerns the introduction of a new silicone (H44) with a different chemical structure (poly-methyl-phenyl-silsesquioxane), compared to those used in preliminary studies [26]. The change is not straightforward, since it affects the ceramic yield, i.e., the amount of silica-based residue with respect to the initial amount of polymer and, more importantly, the formulation of the ceramic residue after treatment in a nitrogen atmosphere. Such treatment yields unprecedented Biosilicate-C composites. The results of an in vitro study determining the cytotoxicity of the materials by evaluating the relative cell viability are presented as preliminary proof of the biocompatibility of these composites.

The assessment of the feasibility of the desired composition by using H44 finally triggers the exploration of further shaping possibilities, exploiting the distinctive gas release below 100 °C and the miscibility with photocurable liquid acrylates for the application of advanced digital light processing.

## 2. Materials and Methods

### 2.1. Starting Materials

Two commercial solid silicone resins, MK and H44 (both from Wacker Chemie GmbH, Munich, Germany) were used as silica precursors. The polymers were expected to achieve a ceramic yield of 84 wt.% (MK) and 53 wt.% (H44) after firing in air, and 84 wt.% (MK) and 72 wt.% (H44) after firing in N_2_ at 1000 °C. The ceramic residue was expected to consist of pure silica (SiO_2_) for treatment in air, or of a SiOC nanocomposite for treatment in nitrogen. Calcium carbonate (CaCO_3_, <10 μm, industrial grade, Bitossi, Vinci, Italy), sodium carbonate (Na_2_CO_3_, <10 μm, reagent grade, Sigma-Aldrich, Germany) and hydrated sodium phosphate (Na_2_HPO_4_·12H_2_O, <10 μm, reagent grade, Sigma-Aldrich, Gillingham, UK) were used as active fillers in a first batch of samples (Table 1). Anhydrous sodium phosphate (Na_4_P_2_O_6_, <10 μm, reagent grade, Sigma-Aldrich, Gillingham, UK) was also used as the source of Na_2_O and P_2_O_5_ in a second series of experiments (Table 2).

### 2.2. Direct Ink Writing of 3D Scaffolds

**Figure 1:**
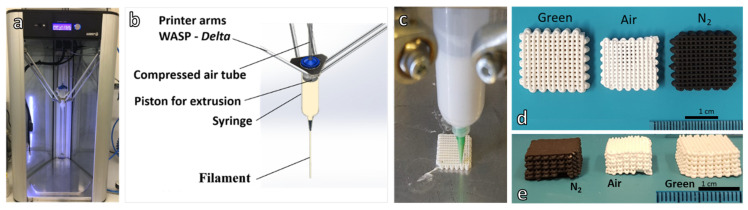
Direct ink writing experiments: (**a**) overall view of the printer; (**b**) scheme of the printing head; (**c**) printing head during operation; (**d**,**e**) examples of MK-based scaffolds in green form, before heat treatment and after treatment in air and N_2_ atmosphere.

MK and H44 silicones were first dissolved in 22.4 vol.% of isopropyl alcohol (Sigma-Aldrich, Gillingham, UK) and then mixed with nano-sized fumed silica (FS; SiO_2_, AEROSIL R106; Evonik, Essen Germany), with an average primary particle size of ~7 nm and BET surface area 220–280 m^2^/g (as specified by the supplier). Fumed silica was used as a rheology modifier (10 wt.% of the total silica content). Fillers yielding CaO, Na_2_O and P_2_O_5_ were added in the second step, after preparation of a homogeneous, agglomerate-free, silicone/fumed silica gel. After mechanical mixing (Argo Lab AM20-D mixer operating at 350 rpm) for 90 min, the silicone-filler pastes were used as inks which were placed into a pressurized cartridge for direct ink writing experiments. Three-dimensional scaffolds were produced from overlapping filaments extruded through a conical nozzle (Nordson EFD, Westlake, OH, USA) with a diameter of 800 μm, utilizing a Delta printer (Delta WASP 2040 Turbo; Wasp, Massa Lombarda, Italy, Figure 1).

After printing, the scaffolds were left for one day at room temperature to evaporate solvent completely. Finally, the scaffolds were fired—in air or in nitrogen—at a heating rate of 1 °C/min, with holding stages at 500 °C for 3 h, and 1000 °C for 1 h. An electric muffle furnace was used for treatment in static air; a tubular furnace was adopted for treatment in flowing N_2_, after vacuum purging. The fired samples were then cooled down to room temperature in the furnace at a rate of 5 °C/min. The firing schedule followed the conditions from a previous investigation [27]. Selected samples prepared with the use of the H44 polymer mixed with anhydrous Na-phosphate were subjected to a pre-firing step at 60 or 75 °C for 24 h, before final thermal treatment.

### 2.3. Digital Light Processing of H44-Based Formulation

The H44 silicone was blended with a commercial photocurable liquid acrylates (acrylate monomers and glycol diacrylate monomers) mixed with phosphine oxide-based photo initiator (Standard Blend (SB), Fun To Do, Alkmaar, The Netherlands). The first step involved only mixing in isopropyl alcohol using the H44/solvent/SB weight ratios of 1/0.5/1. The mixing was performed using a planetary mixer (THINKY ARE-250) for 30 min at a speed of 2000 rpm. In the second step, oxide fillers were added to the liquid blend, in the amounts listed in Table 3, and further mixed in planetary mixer for 30 min.

Homogeneous mixtures were processed using a DLP printer (Original Prusa SL-1, Prusa Research a.s., Prague, Czech Republic) operating in the visible light range (405 nm). The layer thickness was set at 50 µm and the exposure time at 15 s. After printing, the structures were cleaned by flow of compressed air and washing in isopropyl alcohol. To ensure complete hardening, the printed parts were further cured in a UV chamber (365 nm, Robotfactory S.r.l., Mirano, Italy) for 30 min. Finally, the printed parts were transformed to ceramic by the two-step heat treatment in flowing nitrogen: 0.5 °C/min up to 500 °C for 5 h, followed by heating at 2 °C/min up to 1000 °C for 1 h.

### 2.4. Microstructural Characterization

A digital calliper was used to measure the dimensions of all samples after heat treatment. Helium pycnometer (Micromeritics AccuPyc 1330, Norcross, GA, USA) was used to determine the apparent and true density of the highly porous foams and scaffolds. Scanning electron microscopy (SEM, FEI Quanta 200 ESEM, Eindhoven, The Netherlands) equipped with energy dispersive spectroscopy (EDS) was used for the microstructural characterizations of the samples.

Mineralogical analysis was performed on powdered samples by X-ray diffraction (XRD, Bruker AXS D8 Advance, Bruker, Germany), supported by the Match! program package (CRYSTAL IMPACT GbR, Bonn, Germany). Samples were finally subjected to compression tests (Quasar 25, Galdabini, Cardano, Italy), operating at a crosshead speed of 1 mm min^−1^. Each data point represents an average value obtained by testing of at least eight specimens.

### 2.5. In Vitro Cytotoxicity Assay

Stromal cell line ST2, derived from mouse bone marrow, (Deutsche Sammlung von Mikroorganismen und Zellkulturen GmbH, Braunschweig, Germany) was cultured in RPMI 1640 medium (Thermo Scientific, Schwerte, Germany) supplemented with 10% *v*/*v* foetal bovine serum (FBS, Sigma-Aldrich, Taufkirchen, Germany) and 1.0% *v*/*v* penicillin streptomycin (PS, Life Technology, Darmstadt, Germany).

ST2 cells were seeded into 24-well plates with an inoculum ratio of 1 × 10^5^ cells/mL and maintained in an incubator (New Brunswick TM Galaxy^®^ 170 R CO_2_ Incubators, Eppendorf AG, Hamburg, Germany) at 37 °C in a controlled atmosphere of 5.0% CO_2_ for 24 h. MK and H44-based scaffolds, in powder form, were sterilized by heat treatment at 180 °C for 3 h in a furnace (Nabertherm GmbH, Lilienthal, Germany). An amount of 1 g of sterilized powdered samples was added to 10 mL RPMI medium (without cells) and incubated separately for 24 h at 37 °C under the same condition as cells located in 24-well plates. After 24 h, the supernatant was extracted, diluted to 1, 0.1 and 0.01% *w*/*v* concentrations and placed in contact with ST2 cells in 24-well plates and incubated for 48 h. Cells cultured in the medium were used as positive control while cells with RPMI containing 6% *v*/*v* dimethyl sulfoxide (DMSO) and were used as a negative control. Each sample was prepared in triplicate.

WST-8 assay (CCK-8 Kit, Sigma-Aldrich, Germany) was carried out to evaluate cell viability. In total, 1% *v*/*v* WST-8 in colourless cell medium solution was added in each well in the amount of 400 μL and incubated for 3 h. WST-8 solution without cells was incubated at the same time and used as a blank. Later, aliquots of 100 μL from each well were transferred to a 96-well plate for the spectrometry measurements with a microplate reader (PHOmo Elisa reader, Autobio Diagnostics Co. Ltd., Zhengzhou, China) at 450 nm. Each experiment were carried out in triplicate. The cell viability was calculated as follows:

(1) $$\mathrm{Cell}\;\mathrm{Viability}\;\left(\%\right)=\frac{\left(\mathrm{Absorbance}\;\mathrm{of}\;\mathrm{sample}-\mathrm{Absorbance}\;\mathrm{of}\;\mathrm{Blank}\right)}{\left(\mathrm{Absorbance}\;\mathrm{of}\;\mathrm{positive}\;\mathrm{control}-\mathrm{Absorbance}\;\mathrm{of}\;\mathrm{blank}\right)}\times 100$$

After the WST-8 assay, the histochemical staining using haematoxylin and eosin (H&E) was carried out and the samples were examined with light microscope (LM, Primo vert, Carl Zeiss AG, Jena, Germany).

## 3. Results and Discussion

### 3.1. Direct Ink Writing and Firing in Air: Confirmation of Biosilicate^®^-like Composition

**Figure 2:**
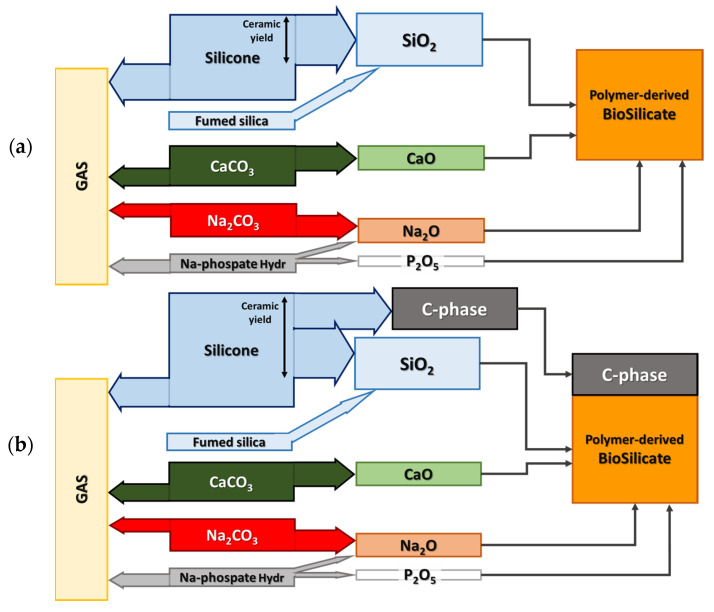
Scheme for the formulation of silicone-based inks aimed at transformation into: (**a**) polymer-derived Biosilicate^®^-like ceramic; (**b**) polymer-derived Biosilicate^®^-like ceramic/C composite.

As shown in Table 1, all formulations for the printing of three-dimensional scaffolds, later fired in air, started from a reference amount of SiO_2_ (10 g) [27]. Fumed silica was introduced as a ‘functional’ filler, to prepare inks with a pseudoplastic behaviour [12], to avoid the viscous collapse of printed structures. This component provided a fixed but minor amount (10 wt.%) of the total silica required to form a Biosilicate^®^-like system; the main contribution (90 wt.%) came from silicones. The increased amount of H44 (17 g for 9 g of SiO_2_), compared to MK (10.71 g for 9 g of SiO_2_), was designed to compensate for its substantially lower ceramic yield. The amounts of other fillers were calibrated according to the oxide balance of Biosilicate^®^ glass-ceramics (Figure 2a) and to the ‘oxide yield’ of the chosen compound. As an example, given the CaO/SiO_2_ proportion in Biosilicate^®^, any 10 g of SiO_2_ had to be accompanied by 4.9 g of CaO, in turn provided by 8.74 g of calcite (CaCO_3,_ undergoing decomposition upon heating). The small amount of P_2_O_5_ (0.82 g of P_2_O_5_ for 10 g of SiO_2_) was provided by hydrated sodium phosphate, which actually yielded part of Na_2_O as well (and the release of water vapour). Sodium carbonate (Na_2_CO_3_) was finally provided the remaining amount of Na_2_O to achieve the characteristic Na_2_O/SiO_2_ proportion in Biosilicate^®^.

**Figure 3:**
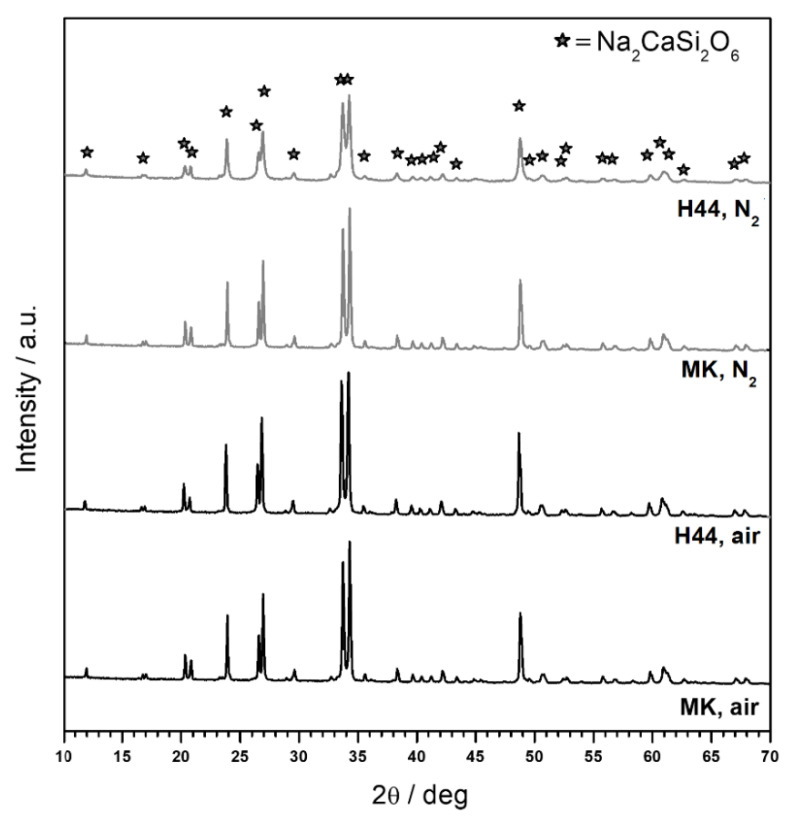
X-ray diffraction analysis of polymer-derived Biosilicate^®^-like scaffolds: comparison of formulations (MK: ink based on MK silicone; H44: ink based on H44 silicone) and firing atmosphere.

The MK silicone-based ink with the formulation used in our previous work [27] acted as a ‘benchmark’ which, after firing, yielded a product with the phase assemblage exactly matching the Biosilicate^®^ glass-ceramics. Figure 3 testifies that after the proper compensation for the lower ceramic yield, H44 could represent a valid alternative after firing at 1100 °C in air. The typical main phase of Biosilicate^®^ glass-ceramics (Na_2_CaSi_2_O_6_, PDF#77-2189) was detected.

**Figure 4:**
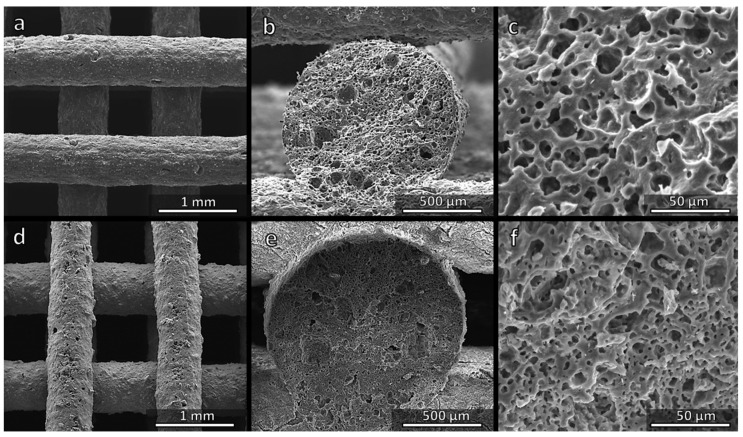
Comparison of the microstructure of Biosilicate^®^-like scaffolds fired in air, from MK (**a**–**c**) and H44 (**d**–**f**) silicone polymers.

Figure 4a shows a regular and crack-free three-dimensional scaffold prepared successfully from the MK-based ink. As observed previously, a sufficient interpenetration of filaments belonging to different layers (Figure 4b) was achieved and, more importantly, the struts were highly porous (Figure 4b,c). The spongy structure (Figure 4c) results from gases released from fillers within the silicone matrix at low temperatures (<500 °C), i.e., before the polymer-to-ceramic conversion of the silicone could take place. More precisely, both hydrated sodium phosphate and Na_2_CO_3_ decompose below 350 °C, releasing water vapour and CO_2_, respectively. A batch for the preparation of 20.62 g of the Biosilicate^®^-like material (calculated for 10 g of SiO_2_) required 31.73 g of the ink (Table 1). The transformation into ceramic corresponded to a weight loss of ~35%, one half of which took place at a low temperature (~8% could be assigned to water vapour and ~9% to CO_2_ from Na_2_CO_3_). A subsequent gas release at higher temperatures did not lead to any cracking, since gases could escape through the pores formed at a lower temperature. In addition, the liquid phase provided by the phosphate component, transformed upon cooling into the glass phase, allowing for stress relaxation.

Replacing MK with H44 resulted in a higher weight loss (~47%), with a significant contribution from the polymer (~22%). Even at a low temperature some contribution from the polymer could not be excluded, since H44 is known to release water vapour as a product of cross-linking reactions from the condensation of hydroxyl groups attached to adjacent siloxane chains [8]. Despite these facts, however, H44-derived scaffolds were not substantially different from those prepared from the MK polymer. Figure 4d confirms the absence of cracks, whereas Figure 4e,f illustrates the formation of ‘spongy’ struts. The surface of struts (Figure 4d) appeared slightly rougher: in the perspective of bone tissue applications, this feature is quite attractive, since it would favour the attachment of cells [28].

**Figure 5:**
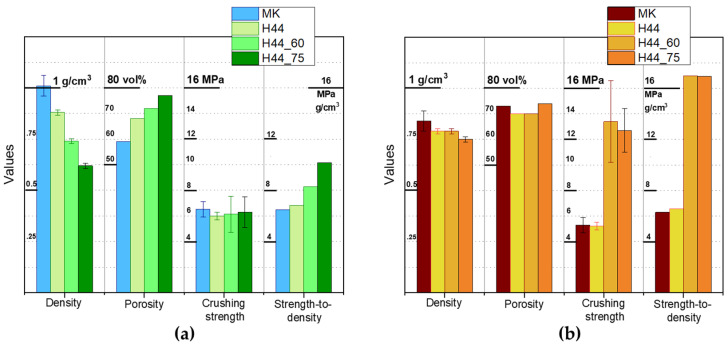
Overview of the properties of polymer-derived Biosilicate^®^-like scaffolds: (**a**) fired in air; (**b**) fired in N_2._ Each data point was obtained by testing at least eight specimens per sample.

The equivalence of MK- and H44-derived scaffolds is further documented by Figure 5a. The density values refer to the geometrical density, while the porosity values correspond to the total porosity, calculated from the geometrical density and true density (~2.7 g/cm^3^), determined by helium pycnometry using powders from crushed samples. The porosity was almost totally open (differences in the order of 2%), given the limited difference between the apparent density (from helium pycnometry of whole samples) and the true density. H44-derived samples were slightly more porous (consistently with the enhanced gas release), with a slightly lower strength. However, the strength-to-density ratio was nearly identical to that of MK-derived samples. In any case, the ratio (>6 MPa·cm^3^/g) was well above the values reported for three-dimensional scaffolds obtained by direct ink writing and sintering of Biosilicate^®^ glass-ceramic powders (4.6 MPa·cm^3^/g) [29].

### 3.2. Direct Ink Writing and Firing in N_2_: Modulation of Biosilicate^®^-like/C Composites

The possibility of replacing MK with H44 was particularly interesting from the perspective of treatment in the nitrogen atmosphere. The polymer-to-ceramic transformation, passing from oxidative (air) to non-oxidative (N_2_) conditions, results in different ceramic yields and different Si–O–C atomic proportions, according to the specific polymer chemistry. Following previous investigations by Scheffler et al. [30], the molecular formulae for MK- and H44-derived SiOC residues (Si_3_O_4.56_C_1.92_ and Si_3_O_4.6_C_8.45_, respectively) were significantly different.

As illustrated by Figure 2b, inks were prepared (Table 1) also to account for different ceramic yields and molecular formulae of the MK and H44 polymers. More precisely, polymer-derived SiOC was intended to consist of a reactive fraction of amorphous SiO_2_, accompanied by C-containing inert phases, as follows [30]:

MK-derived SiOC: Si_3_O_4.56_C_1.92_ ↔ 2.28 SiO_2_ + 0.72 SiC + 1.2 C

H44-derived SiOC: Si_3_O_4.6_C_8.45_ ↔ 2.3 SiO_2_ + 0.7 SiC + 7.75 C

The amounts of fillers were recalibrated according to the content of the amorphous silica; as an example (see Table 1), 10 g of SiO_2_ came from 1 g of fumed silica and 9 g of polymer-derived silica in turn deriving from 14.09 g of MK, instead of 10.71 g of MK used for treatments in air.

The formation of silicon carbide was not actually probable. The presence of fillers, leading to silicate phases, could determine a deviation from the atomic proportions reported above and/or compromise the stability of Si–C bonds [11]. Previous studies on polymer-derived silicate systems fired in N_2_ revealed free carbon as the most prominent C-based phase [11].

Despite the uncertainties on the ceramic conversion of the silicone matrices, in N_2_, the ‘calibration’ of formulations to account for the new firing condition was successful in keeping the phase assemblage substantially unaltered, as shown by Figure 3. No extra crystal phase(s) were detected. The unreacted fraction of the ceramic residues from silicones contributed to the amorphous phase. The amorphous phase resulted in some ‘dilution’ of the Biosilicate^®^-like system, as it was evident from the reduced height of diffraction peaks of Na_2_CaSi_2_O_6_ in the H44-derived samples. This was reasonable: if SiOC transforms into SiO_2_, SiC and C, the H44-based ink was expected to yield a composite with a more abundant C-based extra phase, compared to the Na–Ca silicate. As specified in Table 1, 10 g of SiO_2_ implied an overall mass of oxides of 20.62 g. Out of this, MK yielded (besides 9 g of SiO_2_) 2.84 g of an extra phase (2.84·100%/(2.84 + 20.62) = ~12%), while H44 yielded 7.98 g (7.98·100%/(7.98 + 20.62) = ~28%).

The preparation of carbon-free systems allowed the assessment of the specific contributions of the C-phase to the mechanical properties. Figure 5b confirms the MK- and H44-derived samples were, in terms of their properties, to a great extent equivalent; compared to air treated samples, an increase in the overall porosity (<70%) and a slight decrease in the crushing strength (<6 MPa) were observed, but the strength-to-density ratio remained excellent (>6 MPa·cm^3^/g).

**Figure 6:**
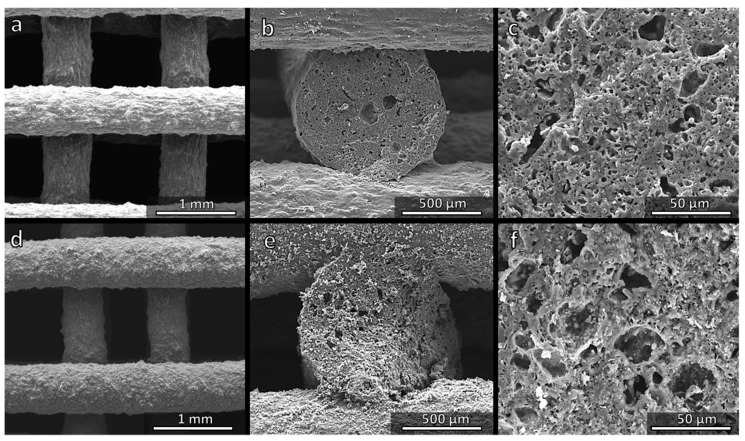
Comparison of the microstructure of Biosilicate^®^-like/C composite scaffolds fired in N_2_, from MK (**a**–**c**) and H44 (**d**–**f**) silicone polymers.

Figure 6a,d confirms the regularity of the structure, the absence of cracks and the roughness of strut surfaces. The cross-sections (Figure 6b,e) confirm the presence of an internal porosity, which was less uniform compared to the samples fired in air (see Figure 4c,f). 

The difference between samples can be attributed, in our opinion, to a series of overlapping effects. The firing in N_2_ does not simply increase the ceramic yield, but also influences the sequence of gas evolution. Additionally, the viscous flow of the phospho-silicate melt, at a high temperature, was likely limited by the presence of the carbon-containing phase.

### 3.3. In Vitro Biocompatibility

**Figure 7:**
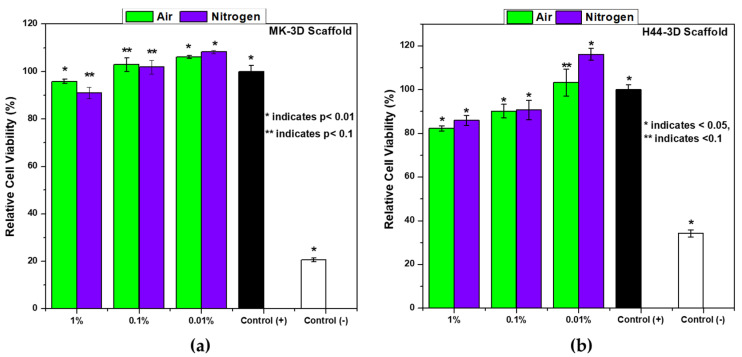
Cell viability of ST-2 cells treated with the supernatant of different concentrations (0.01–1 wt/vol.%) of MK (**a**) and H44-based (**b**) Biosilicate ^®^-like 3D scaffolds after 48 h of incubation.

The results of the ST2 cell viability tests in the presence of extracts of MK and H44-based Biosilicate^®^-like 3D scaffolds fired in air and N_2_ are shown in Figure 7.

The data were evaluated by normalizing the absorbance of positive control as 100% for each sample. Based on the results of a mineralogical analysis, we could expect a successful coupling of a functional phase of pyrolytic carbon with a highly bioactive system, mimicking Biosilicate^®^ glass-ceramics. The obtained biocompatibility results supported the hypothesis and showed that ST2 cell viability increased with the higher dilution of the MK and H44-based 3D scaffolds’ extracts after 48 h of culture. Even though the MK-based scaffolds showed a higher cell viability than the H44-based scaffolds, at the concentration of 1% and 0.1% (Figure 7), all materials fulfilled the EN ISO 10993-5 norm concerning the biological evaluation of medical devices [31]. Moreover, for carbon containing scaffolds (both MK and H44-based), 0.01% *w/v* extracts showed a significantly better cell viability than the positive control group.

The enhanced viability of C-containing composites can be attributed to the lack of reactive oxygen species (ROS) [32,33]. An additional contribution could come from hydrophilicity. When silicone polymers (i.e., MK/H44) are fired at a high temperature (at 1000 °C), in air, a hydrophilic behaviour results from the separation of the organic groups bonded to the siloxane backbone [34]. The hydrophilic character brings the material to be well dispersed in water leading to low direct cell–material interactions which in turn reduce the deposition of the materials on the cell membrane since the materials with a hydrophilic character were able to interact dynamically with the cell environment through water molecules. This phenomenon caused the cytotoxicity [32].

**Figure 8:**
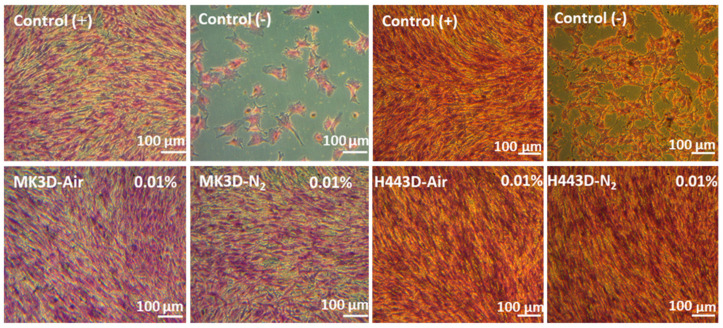
Light microscopy images of H&E-stained ST2 cells cultured with the 0.01% w/v extracts of MK and H44–based Biosilicate ^®^-like 3D scaffolds.

Light microscopy images of H&E-stained ST2 cells cultured with the 0.01% *w*/*v* extracts of MK and H44-based Biosilicate-like scaffolds, and from the positive and negative control, are shown in Figure 8. The cell confluency is correlated with cell viability examined by the WST-8. These results are seen as promising, although a more systematic biological characterization (currently in progress) will be needed.

### 3.4. Direct Ink Writing of H44-Based Pastes: Further Microstructural Modulation

**Figure 9:**
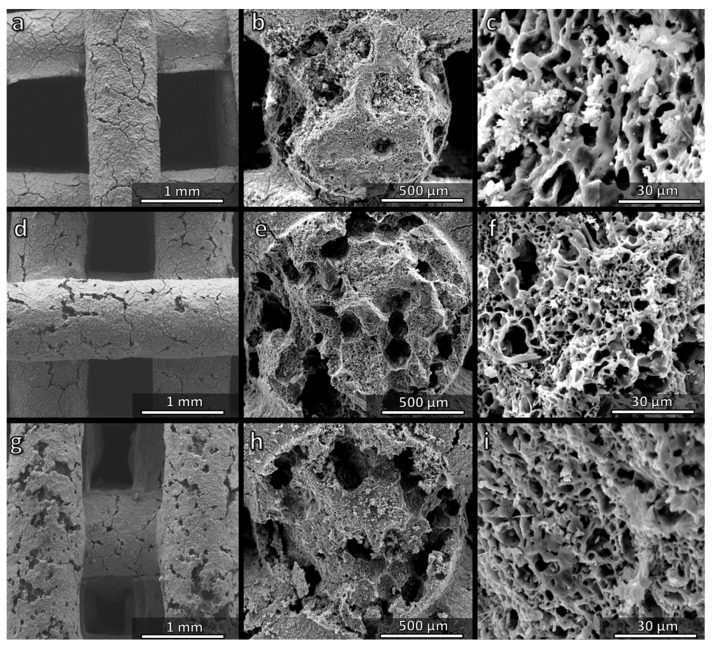
Comparison of the microstructure of Biosilicate^®^-like scaffolds fired in air prepared from H44-based inks comprising anhydrous sodium phosphate: (**a**–**c**) without low temperature heating step; (**d**–**f**) pre-heated at 60 °C; (**g**–**i**) pre-heat at 75 °C.

The possibility of water vapour release at a low temperature from the H44 polymer stimulated the preparation of the second series of inks to be fired in air, in which hydrated sodium phosphate was replaced by anhydrous sodium phosphate, yielding just Na_2_O and P_2_O_5_ (Table 2). Such choice was initially conceived to align the overall weight losses of H44-based inks to those of MK-based inks. However, as a result, the integrity of the scaffold and the homogeneity of the internal porosity were compromised. The only positive result was that the microporosity within the struts was similar to those in the MK-based scaffolds (Figure 9c).

Samples from H44-derived ink comprising anhydrous sodium phosphate were particularly weak, with a multitude of cracks. However, as mentioned above, H44 featured an additional possibility for ‘tuning’ the microstructure, related to the activation of cross-linking reactions at a low temperature. Instead of realizing the cross-linking (with a related gas release) during the heating, additional experiments were carried out involving cross-linking steps at 60 and 75 °C. These experiments resulted in a successful preparation of samples with fewer cracks (Figure 9d,g). The improvement was attributed to more ‘compliant’ structures, i.e., to the enhancement of internal porosity, with large pores, with a diameter up to ~100 µm (Figure 9e,h), which reduced the stiffness and, thus, limited the mechanical stresses arising from the polymer-to-ceramic transformation and crystallization of Na–Ca silicate. Micropores were still present (Figure 9f,i).

According to an X-ray diffraction analysis (not shown), the crosslinking steps at 60 and 75 °C had no impact on the crystallization. Quite surprisingly, the increase in porosity did not cause any degradation of the crushing strength (Figure 5a). The strength-to-density ratio improved significantly, achieving values up to 10 MPa·cm^3^/g. Again, the compliance resulting from an enhanced porosity could have a positive effect in reducing internal stresses. As an example, the presence of residual stresses arising during cooling from different coefficients of thermal expansion of crystalline phases and the glass matrix cannot be excluded, similarly to other glass-ceramic systems [35].

**Figure 10:**
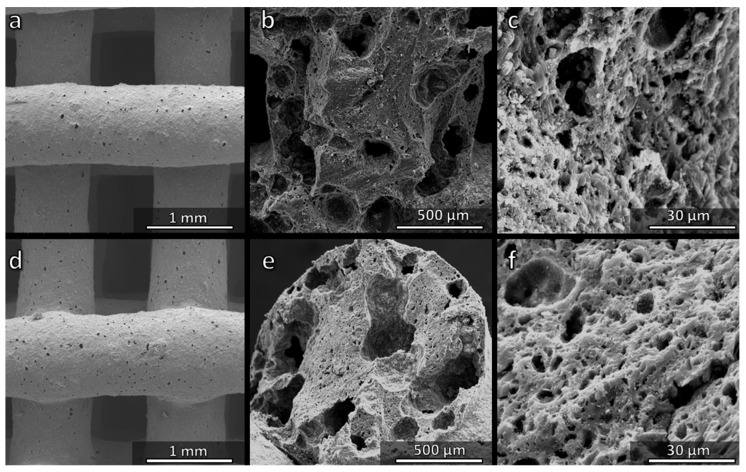
Comparison of the microstructure of Biosilicate^®^-like/C scaffolds fired in N_2_ prepared from H44-based inks comprising anhydrous sodium phosphate: (**a**–**c**) pre-heated at 60 °C; (**d**–**f**) pre-heated at 75 °C.

The heating steps at 60 and 75 °C were finally applied also to scaffolds from H44-based inks comprising anhydrous sodium phosphate, with silicone/filler balance calibrated to prepare a Biosilicate^®^-like/C composite by firing in N_2_. After pre-heating at 60 or 75 °C, the composite samples fired in N_2_ were crack-free (Figure 10a,d). The cross-sections featured large voids (>100 µm, Figure 10b,e) as well as micropores (Figure 10c,f).

Due to the absence of cracks, the samples exhibited a crushing strength exceeding 12 MPa, with the strength-to-density ratio above 16 MPa·cm^3^/g. The observations detected for samples fired in air were probably still valid. Additional contributions to the enhancement of mechanical properties could be attributed to the same synthesis conditions. As observed for other polymer-derived silicate systems previously, the ceramic transformation in an inert atmosphere, which does not involve the strongly exothermic oxidation of Si-CH_3_ bonds may reduce internal stresses [7,8].

### 3.5. H44-Based Formulations: Extension to Digital Light Processing

**Figure 11:**
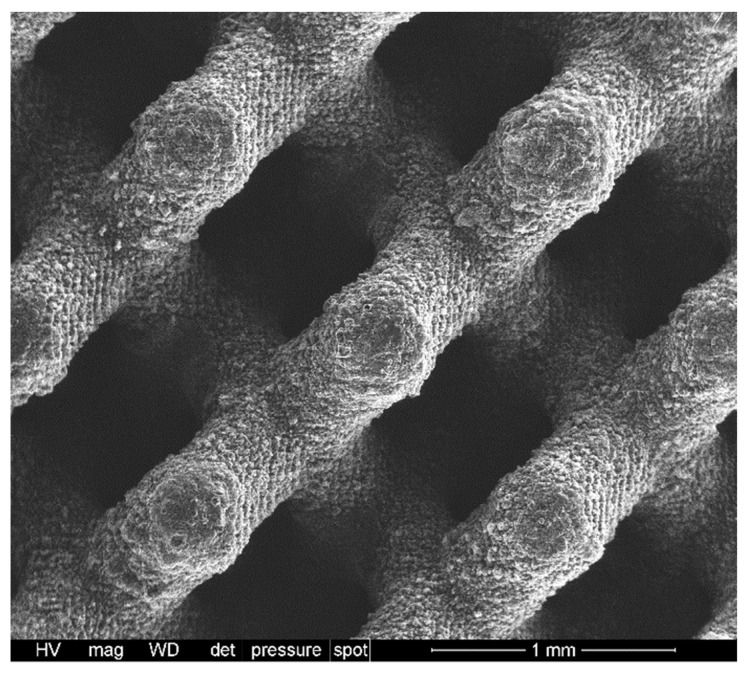
Example of polymer-derived Biosilicate/C scaffold, from DLP of silicone-based photocurable blend.

Compared to MK, H44 has a fundamental advantage in extending the processing options by utilising additive manufacturing technologies. As observed previously [13], H44 may be blended with a photocurable acrylic resin, added with fillers and, finally, subjected to shaping using digital light processing. As shown in Table 3, H44 could be used as the exclusive silica source; the addition of anhydrous sodium phosphate (instead of hydrated sodium phosphate), besides other fillers, was advantageous in promoting the homogeneity of blends. For simplicity, the formulation was tuned to obtain a Biosilicate^®^-like/C composite, in analogy with the scaffolds from direct ink writing. Figure 11 illustrates the achievement of crack-free scaffolds with a diamond cell architecture, after firing in N_2_ at 1100 °C. With a porosity of 77 ± 1 vol.%, the new samples exhibited a crushing strength of 2.5 ± 0.5 MPa, corresponding to a strength-to-density ratio of ~4 MPa·cm^3^/g. Although weaker than scaffolds from direct ink writing, the DLP-derived scaffold still compared well with the three-dimensional scaffolds from Biosilicate^®^ glass-ceramic powders (featuring, as previously observed, a strength-to-density ratio not exceeding 5 MPa·cm^3^/g) [28].

**Figure 12:**
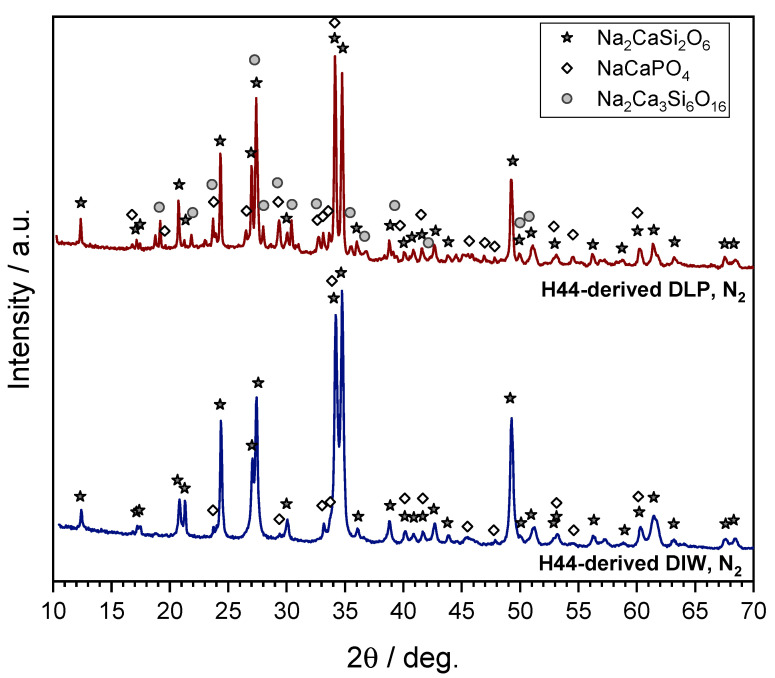
Results of mineralogical analysis of polymer-derived Biosilicate^®^-like/C scaffolds from H44-based mixtures: comparison of scaffolds from DIW and DLP.

The scaffold prepared by digital light processing (DLP) shown in Figure 11 is just a preliminary example. DLP, compared to direct ink writing (DIW), is certainly more attractive for the high flexibility in the design, which will be largely explored in the future. At the present stage, it can be noted that the updated formulation had no negative impact on the phase composition. The results of a detailed mineralogical analysis shown in Figure 12 show that samples from direct ink writing contained, along with Na_2_CaSi_2_O_6_, also traces of a secondary phosphate crystal phase (NaCaPO_4_, PDF#76-1456), similarly to conventional Biosilicate^®^ glass-ceramics prepared by the controlled crystallization of glass [26]. The DLP-prepared samples also featured an additional Na–Ca silicate crystal phase (Na_2_Ca_3_Si_6_O_16_, PDF#77-0386). This, however, does not represent a problem for tissue engineering applications due to the documented biocompatibility and bioactivity of this phase [36,37]. The reduced intensity of diffraction maxima was attributed to the presence of the additional carbon-containing phase. We cannot exclude the formation of the additional C-containing phase from the pyrolysis of the acrylic component. An extensive biological characterization is envisaged also for DLP-derived scaffolds.

## 4. Conclusions

Silicone resins combined with oxide fillers were used for the additive manufacturing of reticulated scaffolds, which, after firing, resembled Biosilicate^®^ glass-ceramics. Changes in the silicone polymer allowed for a fine ‘tuning’ of microstructures. Treatments in nitrogen, after a corresponding modification of the silicone/filler ratios yielded C-containing glass-ceramic composites. The use of a specific silicone (H44) maximized the yield of the secondary phase, which is known to provide extra functionalities. The same silicone enabled the adjustment in the porosity distribution. Combining the revised formulations with treatment in nitrogen, a low-temperature gas release promoted the formation of scaffolds with a hierarchical porosity and an excellent strength-to-density ratio. Moreover, the extracts of MK and H44-based Biosilicate^®^-like scaffolds and carbon containing MK and H44-based Biosilicate^®^-like scaffolds did not exhibit cytotoxicity towards the ST2 stromal cell line. Finally, the selection of suitable silicone facilitated the manufacturing of complex shaped composite scaffolds by digital light processing.

## Figures and Tables

**Table 1:** Batch formulations for polymer-derived Biosilicate scaffolds, by DIW, based on both MK and H44 polymers.

Oxides in Biosilicate^®^ (wt.%)	Oxide Quantities Referred to 10 g of SiO_2_ (g) in Biosilicate^®^		Source Quantities Referred to 10 g of SiO_2_ (g)/Source	
			*Firing in Air*	*Firing in N_2_*
SiO_2_ (48.5%)	10	9	10.71/MK	14.09/MK (2.84 g as extra C-phase)
			17.00/H44	23.58/H44 (7.98 g as extra C-phase)
		1	1/Fumed silica (SiO_2_)	
P_2_O_5_ (4%)	0.82		4.13/Na_2_HPO_4_·12H_2_O	
Na_2_O (23.75%)	4.90	0.72		
		4.18	7.14/Na_2_CO_3_	
CaO (23.75%)	4.90		8.74/CaCO_3_	

**Table 2:** Batch formulations for polymer-derived Biosilicate scaffolds based on H44 and anhydrous sodium phosphate (processed by DIW).

Oxides in Biosilicate^®^ (wt.%)	Oxide Quantities Referred to 10 g of SiO_2_ (g) in Biosilicate^®^		Source Quantities Referred to 10 g of SiO_2_ (g)/Source	
			*Firing in Air*	*Firing in N_2_*
SiO_2_ (48.5%)	10	9	17.00/H44	23.58/H44 (7.98 g as extra C-phase)
		1	1/Fumed silica (SiO_2_)	
P_2_O_5_ (4%)	0.82		1.54/Na_4_P_2_O_6_	
Na_2_O (23.75%)	4.90	0.72		
		4.18	7.14/Na_2_CO_3_	
CaO (23.75%)	4.90		8.74/CaCO_3_	

**Table 3:** Batch formulation for polymer-derived Biosilicate scaffolds shaped by DLP.

Oxides in Biosilicate^®^ (wt.%)	Oxide Quantities Referred to 10 g of SiO_2_ (g) in Biosilicate^®^		Source Quantities Referred to 10 g of SiO_2_ (g)/Source
			*Firing in N_2_*
SiO_2_ (48.5%)	10		26.20/H44 (8.86 g as extra C-phase)
P_2_O_5_ (4%)	0.82		1.54/Na_4_P_2_O_6_
Na_2_O (23.75%)	4.90	0.72	
		4.18	7.14/Na_2_CO_3_
CaO (23.75%)	4.90		8.74/CaCO_3_

## Data Availability

The data presented in this study are available on request from the corresponding author. The data are not publicly available due to privacy restrictions.
